# 2197. Impact of an INtake FOrm for Restricted Medications to (INFORM) better antibiotic use within a long-term acute care hospital system

**DOI:** 10.1093/ofid/ofad500.1819

**Published:** 2023-11-27

**Authors:** Athena L V Hobbs, Jennifer VanCura, Diana A Hobbs, Katherine M Shea

**Affiliations:** Post Acute Medical/Cardinal Health, Memphis, Tennessee; Cardinal Health, Dublin, Ohio; Washington University School of Medicine, St. Louis, Missouri; Cardinal Health, Dublin, Ohio

## Abstract

**Background:**

Long-term Acute Care Hospitals (LTACHs) are uniquely positioned to treat patients with a high severity of illness for a long period of time. Despite this, there are substantial barriers to providing antimicrobial stewardship in this setting including limited resources and lack of patient data from transferring facilities.

**Methods:**

This multi-center retrospective, quasi-experimental study evaluated the use of broad-spectrum antibiotics (BSAbx) over 3 timeframes in 43 LTACHs and rehabs within the Post Acute Medical System. Despite implementation of a restriction policy in June 2018, use remained high for ceftolozane/tazobactam (C/T), ceftazidime/avibactam (CZA), and meropenem/vaborbactam (MEV). In July 2020, the system antimicrobial stewardship committee created an INtake FOrm for Restricted Medications (INFORM) that required submission of pertinent clinical information for each patient receiving one of the target antibiotics as well as systematic review by an ID pharmacist. An electronic version of the INFORM was implemented in April 2022. Study timeframes included baseline (BL: July 2019-June 2020), phase 1 (P1: July 2020-March 2022), and phase 2 (P2: April 2022-March 2023). The primary objective was to determine if there was a difference in the combined use of C/T, CZA, and MEV measured as cost/acute patient day (APD) across the three timeframes. Secondary analyses included post-hoc analyses between these groups, and an evaluation of C/T, CZA, MEV with the addition of imipenem/cilastatin/relebactam (I/C/R).

**Results:**

There was a significant reduction in antimicrobial use across intervention periods (mean cost/APD BL=2.5, P1=1.3, P2=0.8, p=0.045 using ANOVA, Figure 1). Sub-hoc analyses showed a significant difference for BL:P1 (p=0.02) and BL:P2 (p< 0.01), but not for P1:P2 (p=0.6). Even when adding I/C/R to control for any shifts in utilization to this agent after it was approved by the FDA, there was a statistically significant reduction across the intervention periods (p< 0.01 using ANOVA, Figure 2).

Figure 2: Cost/Acute Patient Day for C/T, CZA, MEV vs. I/C/R over time

**Figure:**
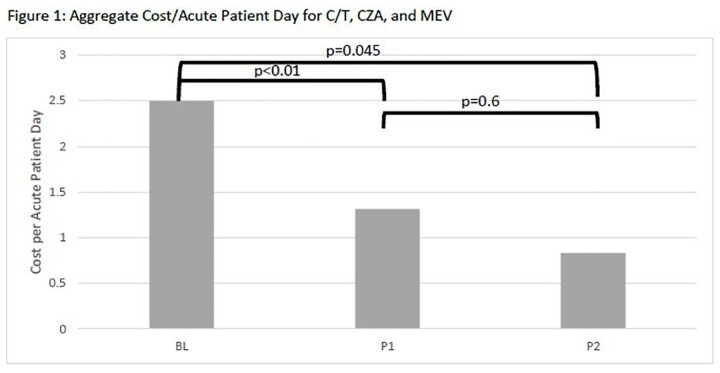

C/T = ceftolozane/tazobactam, CZA = ceftazidime/avibactam, MEV = meropenem/vaborbactam, I/C/R = imipenem/cilastatin/relebactam

**Figure:**
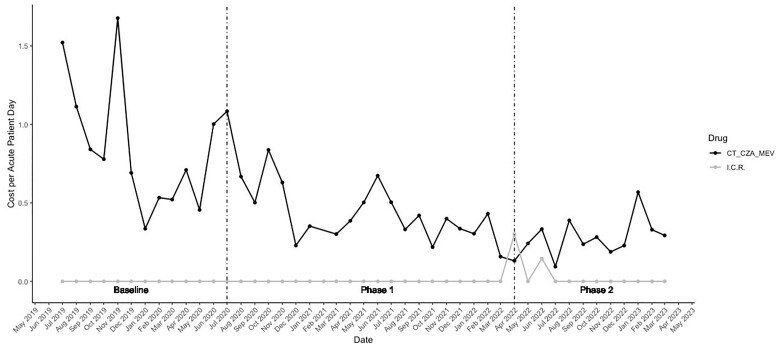

**Conclusion:**

Completion of both the paper and electronic INFORM reduced C/T, CZA, and MEV use from baseline, showing that the systematic collection and review of patient data by an ID pharmacist reduced use of these BSAbx in a system of LTACHs and rehabs.

**Disclosures:**

**All Authors**: No reported disclosures

